# Complete mitochondrial and rDNA complex sequences of important vector species of *Biomphalaria*, obligatory hosts of the human-infecting blood fluke, *Schistosoma mansoni*

**DOI:** 10.1038/s41598-018-25463-z

**Published:** 2018-05-09

**Authors:** Si-Ming Zhang, Lijing Bu, Martina R. Laidemitt, Lijun Lu, Martin W. Mutuku, Gerald M. Mkoji, Eric S. Loker

**Affiliations:** 1 Center for Evolutionary and Theoretical Immunology, Department of Biology, University of New Mexico, Albuquerqu, NM 87131 USA; 2 Centre for Biotechnology Research and Development, Kenya Medical Research Institute (KEMRI), P.O Box, 54840–00200 Nairobi, Kenya; 3 Parasitology Division, Museum of Southwestern Biology, University of New Mexico, Albuquerque, 87131 USA

## Abstract

Using high throughput Illumina sequencing technology, we determined complete sequences for the mitochondrial genome (mitogenome) and nuclear ribosomal DNA (rDNA) complex for three African freshwater snail taxa within the genus *Biomphalaria*, *B. pfeifferi*, *B. sudanica* and *B. choanomphala*, and for two laboratory strains of *B. glabrata* originating from the Neotropics. *Biomphalaria* snails are obligate vectors of the blood fluke *Schistosoma mansoni*, a major etiologic agent of human intestinal schistosomiasis. Our data show that mitogenomes from African and Neotropical *Biomphalaria* are highly conserved. With respect to rDNA, the two internal transcribed spacers (ITS1 and 2) were found to be highly variable whereas the three ribosomal RNA genes (28S, 5.8S and 18S rRNA) exhibited no or very limited variation. Our analyses reveal that the two taxa inhabiting Lake Victoria, *B. sudanica* and *B. choanomphala*, are very similar to one another relative to the similarity either shows to *B. pfeifferi* or *B. glabrata*. This new sequence information may prove useful for developing new markers for snail identification, environmental detection/monitoring purposes or for tracking epidemiology and snail dependencies of *S. mansoni* in endemic areas. It also provides new information pertinent to still unresolved questions in *Biomphalaria* systematics and nomenclature.

## Introduction

Freshwater snails of the genus *Biomphalaria* (Gastropoda, Planorbidae) are obligate intermediate hosts of the digenetic trematode *Schistosoma mansoni*, the causative agent of intestinal schistosomiasis that afflicts over 83 million people across Africa, the Middle East, and South America^1^. In sub-Saharan Africa, where >90% of all cases of *S. mansoni* now occur, the Lake Victoria basin remains a hyperendemic region of transmission. A recent large-scale survey of children in schools around Lake Victoria revealed that prevalence of *S. mansoni* infection in some schools reached up to 90%^2^. Three *Biomphalaria* taxa, *B. pfeifferi*, *B. sudanica* and *B. choanomphala*, vectors of *S. mansoni* in and around the lake, have been found to play a vital role in transmission of schistosomiasis in the region. Although the status of the nomenclature for *B. sudanica* and *B. choanomphala* remains contentious, a point we will return to later in the paper, for the sake of convenience, we use throughout the paper the species names traditionally applied to these taxa^3,4^.

**Figure 1:**
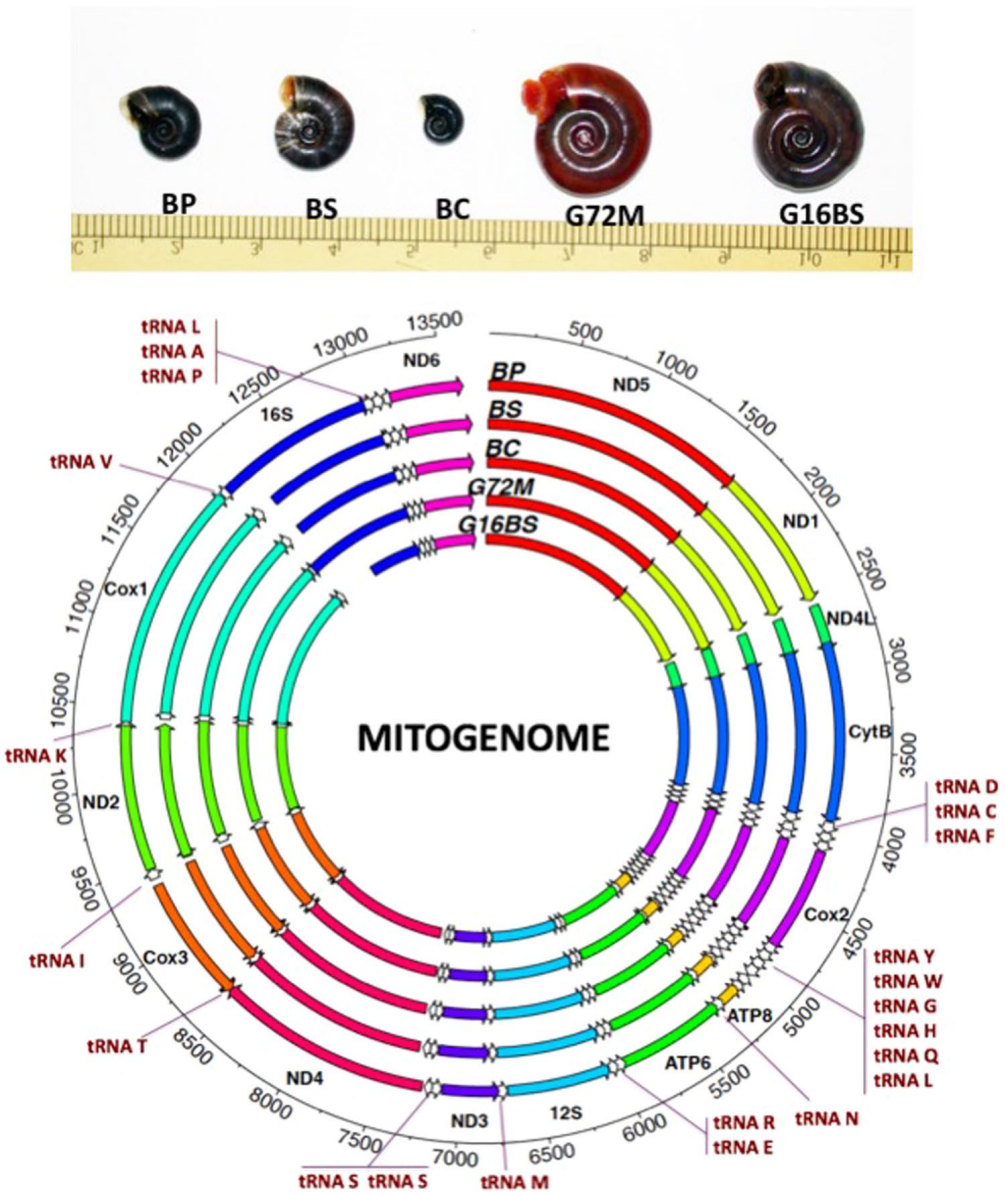
(**A**) Showing the relative size of the five snail specimens. BP: *B. pfeifferi*; BS: *B*. *sudanica*; BC: *B. choanomphala*; G72M: G72 M line *B. glabrata*; G16B: G16 BS90 *B. glabrata*. ND: NADH dehydrogenase subunit; CytB: cytochrome b; Cox: cytochrome c oxidase subunit. ATP: ATP synthase F0 subunit: 12S: small subunit ribosomal RNA; 16S: large subunit ribosomal RNA; Please note that the abbreviations are applied throughout the paper. (**B**) Showing locations of all genes in the mitogenomes. The detailed information for each gene is presented in Supplementary Dataset Table 1.

*Biomphalaria pfeifferi* (Krauss, 1848) is characteristically associated with streams, ponds, reservoirs and irrigation ditches^4–8^. Many streams in which *B. pfeifferi* resides directly connect to Lake Victoria. *B*. *sudanica* and *B. choanomphala* are lacustrine and generally considered to be responsible for the majority of transmission of *S. mansoni* in Lake Victoria^4^. *B. sudanica* is found in swampy areas along the shoreline of the lake whereas *B. choanomphala* is usually found in deeper water (down to 40 m, occurring hundreds of meters from shore), though it can also be collected from the shoreline in some locations^4,9–12^. Although anatomical details of the soft parts of *B. sudanica* and *B. choanomphala* are similar, the two taxa differ in shell size and shape. *B. sudanica* has a relatively flat shell 1.0–1.8 cm in diameter characterized by a flat apical surface and broad and shallow umbilical region. In contrast, the shell of *B. choanomphala* is smaller in diameter (0.5–0.8 cm) and relatively deeper, and shows angulations on the whorls of both upper and lower surfaces (Fig. 1A). Both taxa are susceptible to experimental infections^12,13^ (Mutuku, personal communication) and are found naturally infected with *S. mansoni*^9,14,15^. The question of whether the two taxa should be considered as separate species, subspecies or merely eco-phenotypes has been, and is still, actively discussed^9,15,16^. Standley *et al*.^10^ analyzed mitochondrial CoxI (mtCoxI) and mt16S sequences from *Biomphalaria* collected from Lake Victoria and proposed that *B. sudanica* and *B. choanomphala* should be considered as two subspecies, *B. choanomphala sudanica* and *B. choanomphala choanomphala*, respectively (see Discussion for further consideration).

Molecular analyses based on mitochondrial genome (mitogenome) and nuclear ribosomal DNA (rDNA) have been widely used for identification of species and populations, and for studies of population genetics, evolutionary biology and phylogenetics^17–19^. There are 34 described species of genus *Biomphalaria*, 12 from Africa and 22 from the Americas^4,20–22^. Of the 34 species, eighteen species are known or suspected intermediate hosts for *S. mansoni*^22^. Mitogenomes have been reported only for two South American *Biomphalaria* species, *B. glabrata* and *B. tenagophila*^23,24^, but none from African species.

We used the Illumina platform to sequence complete mitogenomes and rDNA gene complexes for three African taxa, *B. pfeifferi*, *B. sudanica* and *B. choanomphala*. In addition, we also sequenced the mitogenomes of two highly inbred lines of *B. glabrata* Say, 1818, the G72 M line and G16 BS90 lines, which were recently developed in our laboratory. G72 M line and G16 BS90 were derived from two *B. glabrata* strains commonly known M line and BS90 snails, respectively^25,26^. *B*. *glabrata*, the most important species for transmission of schistosomiasis in the New World, was widely adopted over half a century ago as a model snail host for schistosome parasites. Several laboratory strains of *B. glabrata* have since been developed^25–29^. The genome sequence^30^, a linkage map^31^, and complete mitogenome^24^ have been documented for *B. glabrata*. In this study, our goal is to examine the two inbred strains of *B. glabrata* and the three African *Biomphalaria* taxa to assess the extent to which the mitogenomes of Old and New World representatives differ, and how this compares to the extent of differences noted between the taxa *B. sudanica* and *B. choanomphala* from the Lake Victoria region.

## Results

### Mitogenomes

Complete mitogenomes for *B. pfeifferi*, *B. sudanica*, *B*. *choanomphala*, and G72M and G16BS90 lines of *B*. *glabrata* were determined and submitted to GenBank under access numbers MG431962, MG431963, MG431964, MG431965, and MG431966, respectively. All mitogenomes possessed the 37 genes typical of animal mitogenomes, comprising 13 protein-coding genes (PCG), two ribosomal RNA genes (12S rRNA and 16S rRNA), and 22 tRNA genes. The order and orientation of all genes were identical for the five mitogenomes. Differences were found in the length of some genes (Fig. 1B, Supplementary Dataset 1).

Nucleotide composition analysis indicated the mitogenomes were biased towards high A + T content, ranging from 74.68% (G72M) to 76.64% (*B. pfeifferi*). Base composition, measured by the AT-skew and GC-skew, i.e., asymmetry in nucleotide composition, showed AT and GC skews are at the range of −0.11 to −012 and 0.11 to 0.12, respectively, suggesting the preference for A and G nucleotides, a widespread characteristic of animal mtDNAs^32^. Detailed information for nucleotide composition and AT and GC skews in individual genes is provided in the Supplementary Dataset 2.

### Genetic analyses

**Figure 2:**
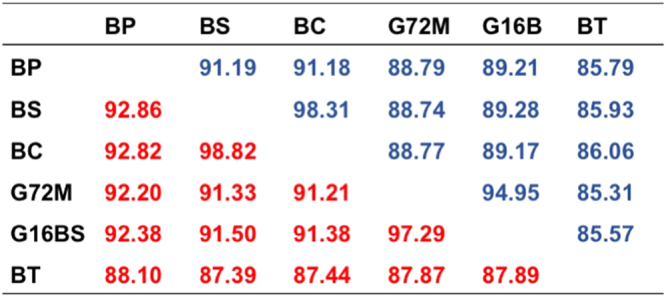
Percent identity matrix of mitogenomes. Blue and red colors show percent identity at nt and aa levels, respectively. At the nt level, whole mitogenome sequences were used. At the aa level, gene products for a given PCG were aligned and trimmed, then all 13 PCGs from a given mitogenome were combined.

**Figure 3:**
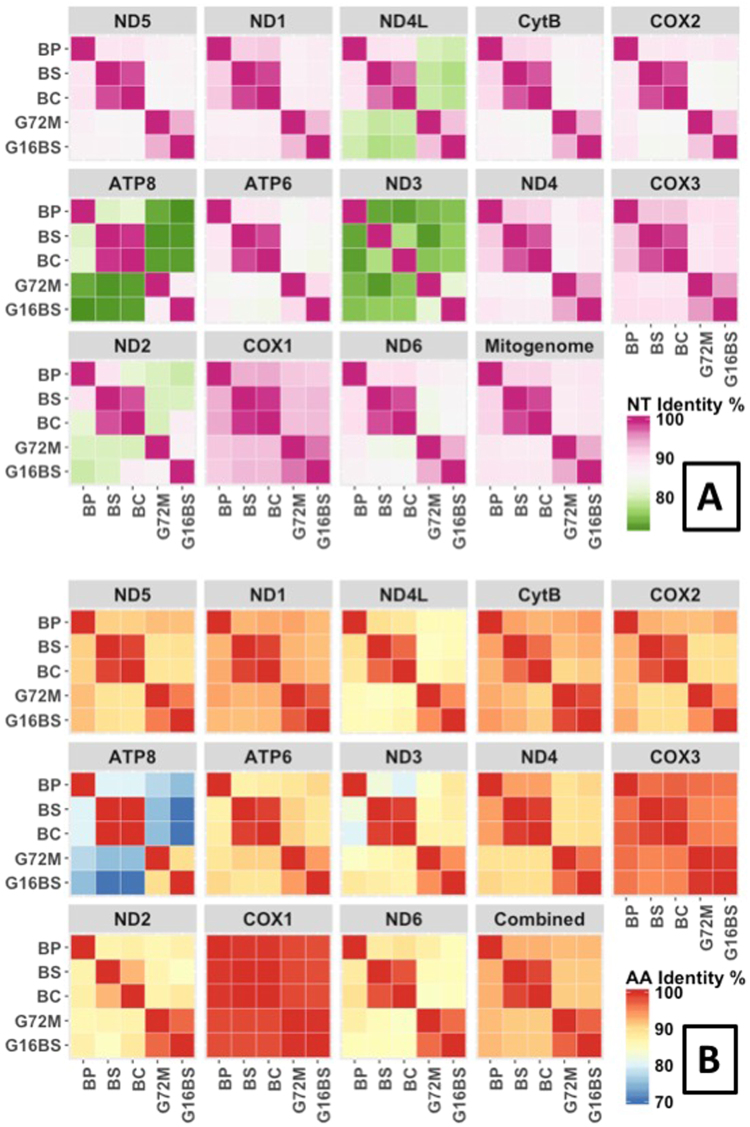
Heat maps showing pairwise comparison of 13 PCGs and complete mitogenome at the nt level (**A**) and gene products of all 13 PCG combined at the aa level (**B**).

**Figure 4:**
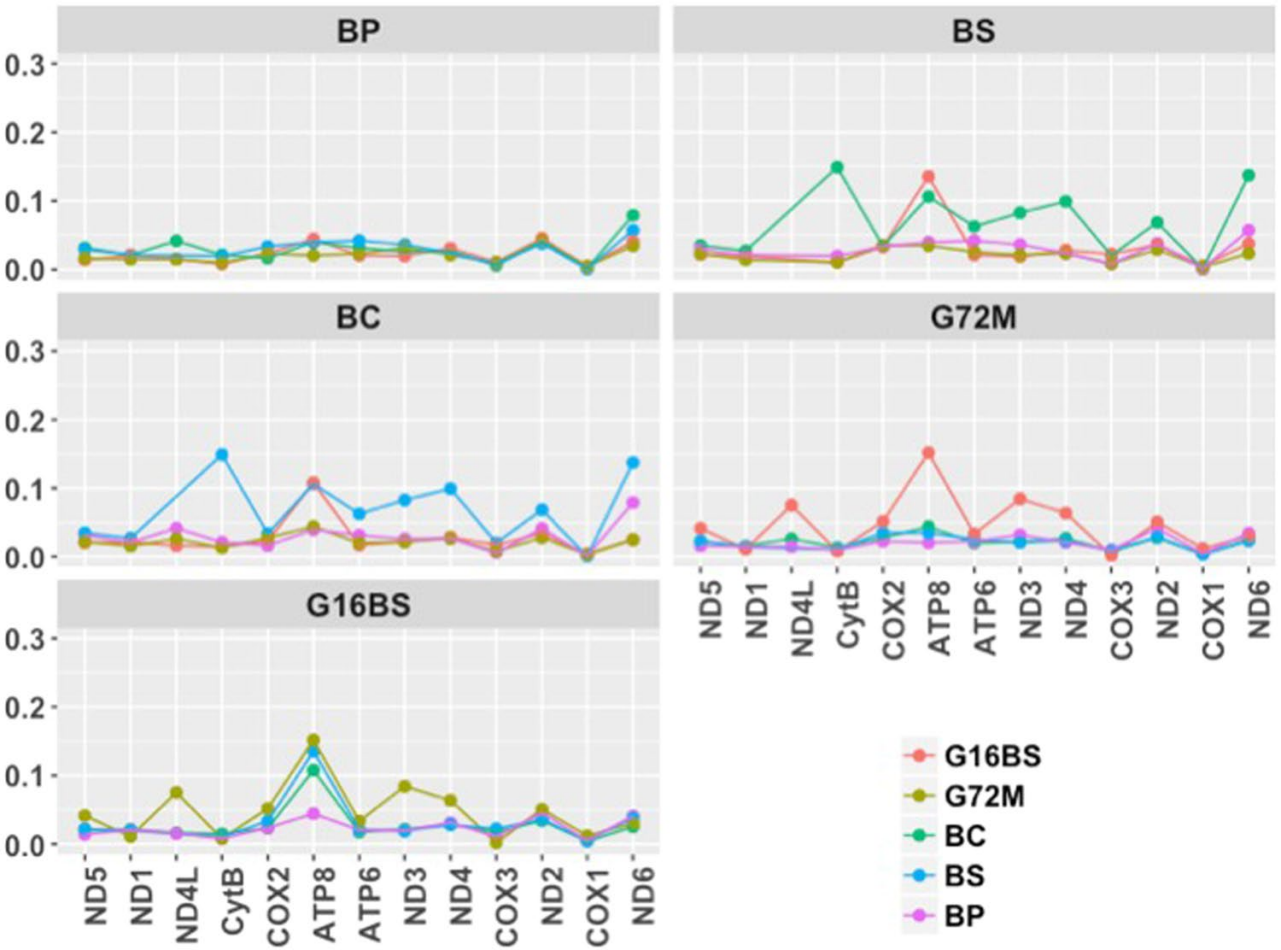
Heat-maps showing the Ka/Ks ratios for pairwise comparison of all 13 PCGs.

The percent identity matrix was generated at the nucleotide (nt) level for the entire mitogenome and at the amino acid (aa) level for gene products of all 13 PCG combined (Fig. 2). It is remarkable that identity between *B. sudanica* and *B. choanomphala* at the nt and aa level is 98.31% and 98.82%, respectively, much higher than that between two strains of *B. glabrata* (94.95% for nt and 97.27% for aa). After including the reported mitogenome of *B. tenagophila* (accession no: EF433576) for comparative analysis, it was found that *B. tenagophila* has the lowest identity with the *Biomphalaria* species included in this study, 85.31–86.06% at the nt level, and 87.39–88.10% at the aa level. Further analyses of individual PCG revealed relatively high variation in ATP8 and ND3, whereas CoxI showed very little variation (Fig. 3A,B). To provide a view of the evolutionary forces acting on mitogenomes of *Biomphalaria*, Ka/Ks ratios generated from individual PCG are provided in Fig. 4. All ratios were found to be less than 1, suggestive of purifying selection acting on all protein coding genes.

### Nuclear rDNA

**Figure 5:**
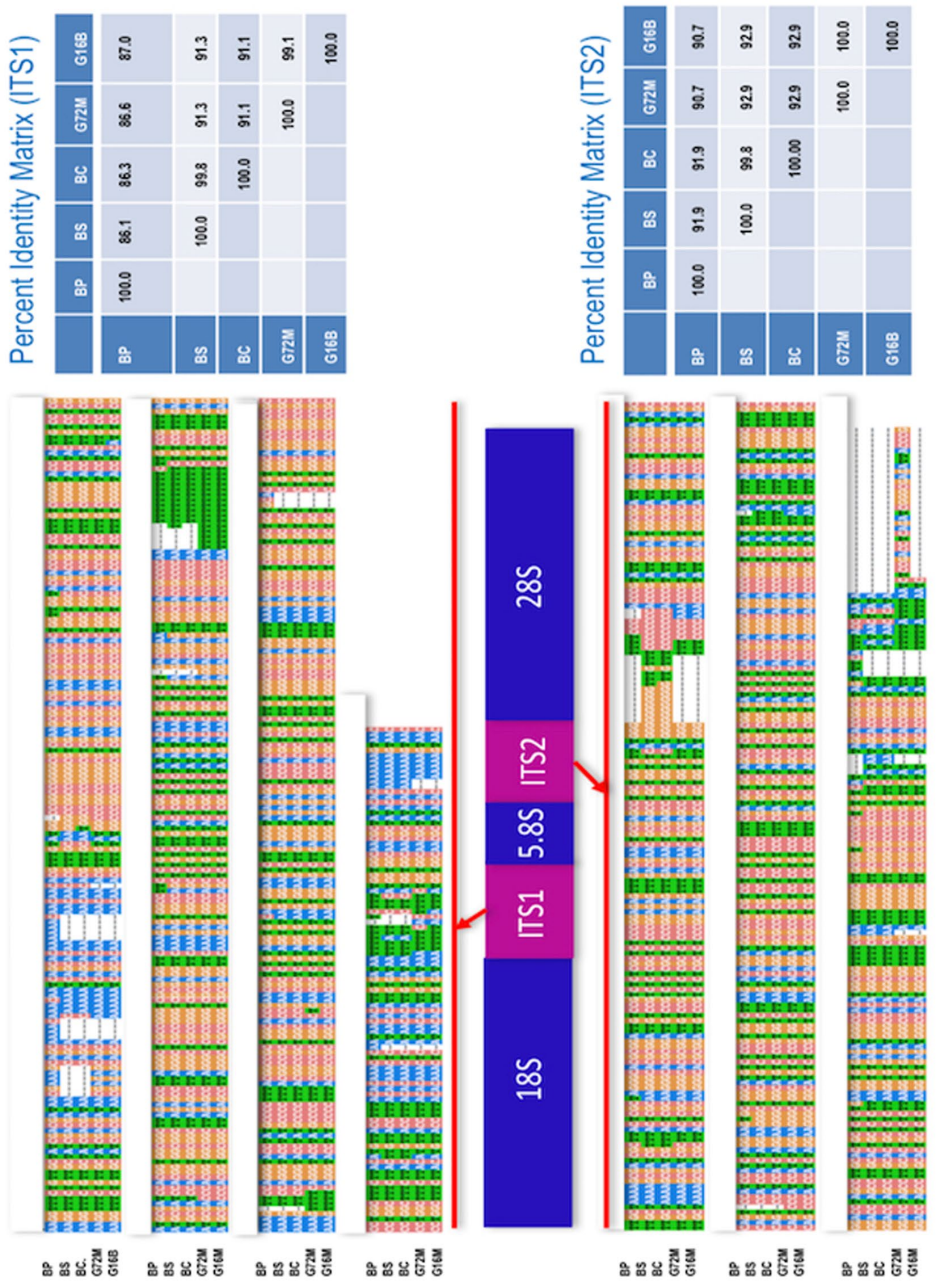
Gene segment of rDNA cassette. Alignments of ITS1 and 2 sequences are shown and percent identity matrixes are provided on the right side.

GenBank accession numbers of rDNA sequences for *B. pfeifferi*, *B. sudanica*, *B. choanomphala*, G72 M line and G16BS90 *B. glabrata* are MG461588, MG461589, MG461590, MG461591, and MG461592, respectively. The sequences of 18S and 5.8S were found to be identical for all five specimens. Minor variation was noted in the 28S region that exhibited overall nt identity at a range of 99.76–100.00%. Differences in 28S sequence were not found between *B. sudanica* and *B. choanomphala* (Supplementary Figure 1). By contrast and not surprisingly, the internal transcribed spacer region 1 (ITS1) and 2 regions exhibited more variation. Figure 5 shows the sequence variations and percent identity of ITS1 and 2 among the five rDNA sequences.

### Phylogenetic analyses

**Figure 6:**
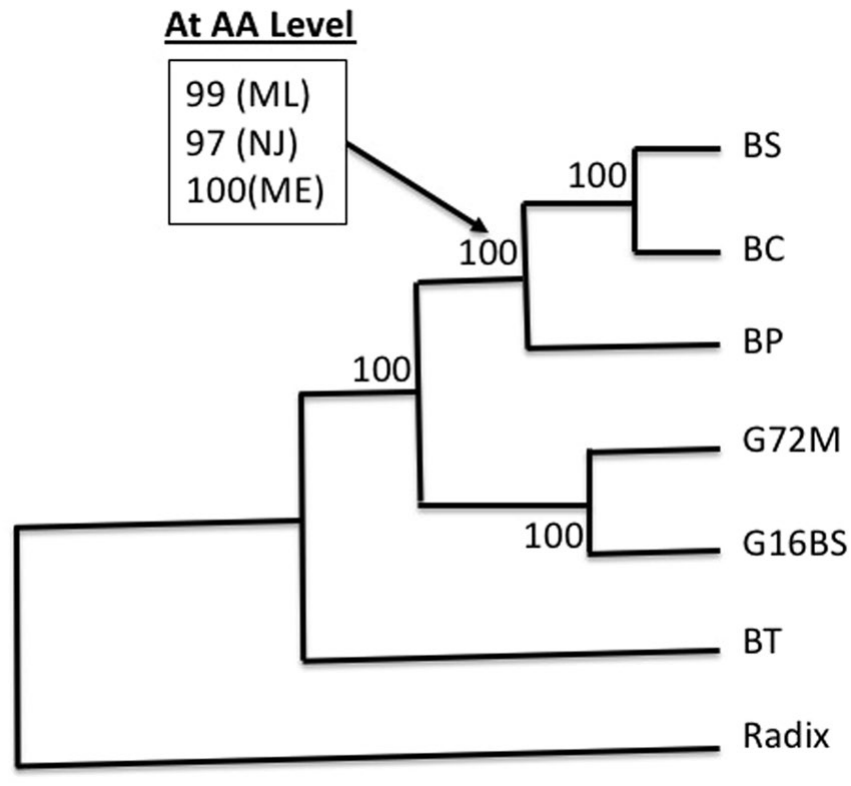
A consensus phylogenetic tree was constructed using whole mtDNA nucleotide sequence and aa sequences of all 13 PCG combined. Three methods, ML, NJ and ME, were used to build the tree. For the whole mtDNA sequences, the three trees are identical in terms of topology and bootstrap values (all with 100). For aa sequences, the topology is exactly the same as that was built by nt sequences. The bootstrap values are almost the same except for one node that is highlighted in the box. GenBank accession numbers for *B. tenagophila* (BT) and *Radix auricularia* are EF433576 and NC_026538, respectively. Please note that due to gene re-arrangement, the gene order of *Physella acuta* (Physidae) is different from that of *Biomphalaria*. So mitogenome of *Radix auricularia* (Lymnaeidae) was used as outgroup.

Using complete mitogenome nt, and aa sequences of all 13 PCG combined, three phylogenetic methods, Maximum Likelihood (ML), Neighbor-Joining (NJ) and Minimum Evolution (ME), were applied. The topology and bootstrap values of all trees based on nt sequence were essentially the same (Fig. 6). For trees based on aa sequences, topology of the three trees was identical to that obtained using the nt sequences, and only slight differences of bootstrap value were found among the three trees. From the trees, it can be seen that the three African species form a clade that is separated from *B. glabrata*. Among the two American species, *B. glabrata* is more closely related to African species than *B*. *tenagophila*.

In addition, we also used the three phylogenetic methods to examine relationships using ITS1, ITS2 and the two ITS combined (Supplementary Figure 2) but as expected, because of the variability of these sequences, they are not as useful for discriminating among species as whole mitogenome sequences.

## Discussion

This study presents complete mitogenome and rDNA sequence for *Biomphalaria* species that play a key role in vectoring *S. mansoni*. *B. pfeifferi* and *B. glabrata* are the two most important intermediate host species for *S. mansoni*, in the Afrotropical and Neotropical regions, respectively. *B. sudanica* and *B. choanomphala* are responsible for transmission of schistosomiasis in Lake Victoria, one of the world’s great hyperendemic schistosomiasis regions. The study provides useful genetic markers for snail diagnostics and systematics, and for schistosome epidemiology.

Gene arrangements reported here are identical to those found in three *Biomphalaria* mitogenomes previously reported, for two strains of *B. glabrata*^24^ and for *B. tenagophila*^23^. This suggests that gene order in mitogenomes of *Biomphalaria* from the Old World (*B. pfeifferi*, *B. sudanica* and *B. choanomphala*) and New World (*B*. *glabrata* and *B. tenagophila*) are highly conserved.

There are five families proposed for the gastropod Order Hygrophila: Acroloxidae, Chilinoidae, Planorbidae, Lymnaeidae and Physidae^33^. So far complete mitogenomes are documented only for representatives of the Planorbidae^23,24^, Lymnaeidae^34^, and Physidae^35^. The order of mitochondrial genes in the Planorbidae to which *Biomphalaria* belongs is similar to the Lymnaeidae except for differences in location of a few tRNAs^23,34^, whereas gene order is quite different in the Physidae^35^ (for more information about the comparative mitogenome gene orders of the gastropod, please see articles)^35,36^. It has been noted that gastropods display an unusually large variety of gene orders among their mitogenomes^36^. Further studies are needed to characterize more complete gastropod mitogenomes, which will shed light on their evolution and diversification, and at the same time, provide more genetic markers for population genetics, evolutionary and phylogenetic studies. Our study also provides additional rDNA sequence data, thereby adding further genetic characterization of more snail species involved in transmission of human schistosomiasis.

Our analyses based on the mitochondrial DNA and nuclear rDNA sequences lead to the following conclusions: (1) Diversity within the species *B. glabrata* is relatively high; (2) Variation between the two Neotropical species is higher than among the Afrotropical species we examined; and (3) Data are consistent with *B. sudanica* and *B. choanomphala* being eco-phenotypes of the same species. Further explanations are provided below.

For *B. glabrata*, four mitogenomes from different strains are now available, two (M line and 1742) reported a decade ago^24^ and two (G72M and G16BS) presented in this study. The 1742 strain was from the Natural History Museum, London, UK^37^, and the M line strain was maintained at University of New Mexico (UNM), USA^24^. Both were originally derived from early crosses between albino Brazilian strain, resistant to Puerto Rican *S. mansoni*, and pigmented Puerto Rican snails, susceptible to the same parasite^25^. The G72 M line was derived from the UNM M line colony by continually selecting snails derived from self-fertilization. Not surprisingly, M line (accession no: AY380567), 1742 (AY380531) and G72M line share high genetic similarity (99.87–99.93% nt identity). The pigmented BS90 strain, a schistosome-resistant strain, also referred to frequently as the Salvador strain, was isolated in Salvador, Brazil^26^. G16BS90 was derived from the BS90 strain by repeatedly selecting snails by self-fertilization. It is notable that the divergence between G16BS90 and the other three strains of *B. glabrata* is high (94.95%). This supports earlier observations of high genetic variation within *B. glabrata*^22,38^.

Because *Biomphalaria* are vectors for *S. mansoni*, the origin and diversification of the genus has attracted much attention. An early hypothesis suggested that the origin of *Biomphalaria* was in Gondwanaland as much as 100 million years ago (MYA), and as a consequence, *Biomphalaria* snails were already present on what became Africa and South America as they rafted apart^39,40^. A number of molecular investigations using allozymes, PCR-based fragments of mtDNA or partial rDNA sequences, have since suggested that *Biomphalaria* snails originated in South America, and later dispersed to Africa less than 3 MYA and gave rise to the African species^21,22,37,38,41–43^. Our phylogenetic analyses based on whole mitogenome sequences demonstrated that *B. glabrata*, not *B. tenagophila*, is closely related to the group of African species, adding additional evidence suggesting that African *Biomphalaria* came from the New World’s species, likely from a *B. glabrata*-like ancestor^22,37,38^.

Identification of *Biomphalaria* species is challenging because shell features are often highly variable and can even be confused with shells of other genera like *Helisoma*^44^. Anatomical characteristics, often revealed only by painstaking dissections of specially prepared materials, require experience, patience and practice to discern^13,45,46^. Nonetheless, reference to both shell and anatomical features in conjunction with provision of an ever-increasing array of molecular data has provided an improved and workable overall systematic framework for *Biomphalaria*. However, significant conundrums remain, among them the relationships between, and naming considerations for *B. sudanica* and *B. choanomphala* from Lake Victoria. This issue is of interest because, as previously noted, the shells of the two taxa are quite distinct, and because both play key roles in transmission of *S. mansoni* in the lake. Our phylogenetic analyses indicated the two taxa are closely related, consistent with representing the same species, as evidenced by the high sequence identity in their mitogenomes (98.31% nt identity), higher than that observed among different strains of *B*. *glabrata* (94.95% nt identity). An allozyme-based study also supported a close relationship between the two taxa but also showed some evidence of separate gene pools between them^42^. DeJong *et al*.^22^ using mt16S and ITS1 and 2 sequences regarded *B. choanomphala* as a lacustrine form of *B. sudanica*. Based on restriction enzyme digestion of ITS1 and mtCoxI sequence, Standley *et al*.^10,11^ noted that specimens with both classic *B. choanomphala* and *B. sudanica* shell forms occurred among various clades in a CoxI tree. They noted that habitat predicted shell morphology and suggested snails exemplifying the two shell forms were ecophenotypes of the same species. They did not differentiate between the two ecophenotypes in their study of the population genetics of *Biomphalaria* in Lake Victoria^11^.

With respect to what to formally call the *Biomphalaria* snails inhabiting Lake Victoria, Standley *et al*.^10^ reasonably proposed that the name *B*. *choanomphala* should take priority based on the description of this species from the lake by Martens^47^. They proposed the two taxa should then be considered as *B. c. choanomphala* and *B. c. sudanica*. They argued that *B. sudanica* from other localities in Africa is likely a separate species, based on the genetic distance observed between the Lake Victoria samples and those from Genbank, whereas *B*. *choanomphala* is considered endemic to Lake Victoria^10^.

Martens first described *B. sudanica* in 1870 with the type locality given as the Bahr el Ghazal at Meshra el Req, in what is now South Sudan. Nine years later (1879), he described *B. choanomphala* based on specimens collected from the southwest shore of Lake Victoria in Tanzania^4,48^. In 1890, he re-described *B. sudanica* and *B. choanomphala* both collected from an area called Nyansa (present day West Kenya) of Lake Victoria, implying that *B. sudanica* in Lake Victoria should be considered as the same species he first described from the South Sudan type locality. Key to the decision as to what name to apply to the Lake Victoria specimens is whether *B. sudanica*, given the temporal priority of the name, from elsewhere in Africa but most especially from the original type locality, is genetically distinct from *Biomphalaria* specimens from Lake Victoria. A phylogenetic analysis based on sequence data currently available in GenBank does not provide bootstrap support for differentiating *B. sudanica* from Lake Victoria from *B. sudanica* from other locations (Supplementary Figure 3), but more morphological and molecular data, particularly for *B. sudanica* from South Sudan or Sudan, are needed. If *B. sudanica* from the type locality is found to be genetically similar to those from the lake, then this name would have precedence and *Biomphalaria* from Lake Victoria might best be called *B. sudanica sudanica* and *B. sudanica choanomphala* or *B. sudanica* (one species with two ecophenotypes). Also, although our data support the finding by Standley *et al*.^10^ that *B. sudanica* and *B. choanomphala* from the lake are genetically very similar, it is not clear how commonly gene flow may occur within and between the two ecophenotypes, and whether this could ever significantly influence their respective levels of susceptibility to *S. mansoni*. Here it should be noted that *Biomphalaria* in the lake must respond to selective pressures imposed by many different trematode species, along with other biotic and physical factors, and these factors are bound to differ between the shoreline habitats frequented by *B. sudanica* and the deeper waters usually occupied by *B. choanomphala*.

Finally, we note that mtDNA and rDNA from both snails and schistosomes have proven to have considerable utility as source material for the development of marker sequences of potential use in various kinds of diagnostic or identification assays^49^. We note that as schistosomiasis control efforts enter phases where surveillance and monitoring efforts of snail populations become more critical, having reliable tools for proper identification of the snail species present and defining their role in transmission will be critical. Provision of additional sequence data to aid this effort is another potential benefit of this work.

## Materials and Methods

### Specimens

All snail specimens sequenced were laboratory-reared at the Center for Evolutionary and Theoretical Immunology, Department of Biology, at the University of New Mexico (UNM). Founding stocks of *B. pfeifferi*, *B. sudanica* and *B. choanomphala* were originally collected in western Kenya, and have been maintained for 5, 4, and 0.5 years, respectively. This project was undertaken with approval of Kenya’s National Commission for Science, Technology, and Innovation (permit number NACOSTI/P/15/9609/4270), National Environment Management Authority (NEMA/AGR/46/2014) and the Kenya Wildlife Service (permit #0004754). *B*. *pfeifferi* was collected from Asao stream (00°19′5.50′′S, 35°0′24.99′′E). *B. sudanica* and *B. choanomphala* were collected from Lake Victoria: *B. sudanica* from the car-wash site in Kisumu, Kenya (00°05′45.00′′S, 34°44′57.69′′E) and *B. choanomphala* from Usenge Beach (00°3′42.00′′S, 34°2′37.00′′E). The M line G72 line and the BS-90 G16 lines of *B. glabrata* snails have been generated through 72 and 16 generations of selfing, respectively, the highly inbred lines that are used for genetic studies of snail resistance to schistosomes. The founding M line *B. glabrata* snails that are susceptible to *S. mansoni* were originally acquired from Dr. C. S. Richards and have been maintained in the lab for at least 40 years. The BS-90 snails, resistant to *S. mansoni*, were acquired from Dr. Lobato Paraense in the late 1980’s and were originally obtained from him from natural habitats in Salvador, Brazil^26^.

### Extraction of DNA

The shell of each snail was removed and the intact whole body was rinsed and then ground to a fine powder using mortar and pestle in liquid nitrogen. The powder was transferred to 1.5 ml tubes for subsequent DNA extraction.

Two methods were used for DNA extraction, depending on the use to which the samples were being put. For *B. pfeifferi*, *B. sudanica* and *B. choanomphala*, we applied a mtDNA enrichment method typically used for mammalian cells^50^. This method was originally developed based on the notion that mtDNA in a eukaryotic cell is comparable to plasmids in a bacterium in terms of size and form. Therefore, a Miniprep kit (Qiagen) normally used for extraction of plasmid DNAs from bacteria was used to extract mtDNA from snail cells.

For G72 M line and G16 BS90 *B. glabrata*, we used the CTAB method^51^ rather than the mtDNA enrichment method as described above. The CTAB method can extract all cellular DNAs (nuclear DNA and mtDNA), which can be subsequently sequenced by next generation sequencing. In this study, our main interest is in the mitogenomes. So we conducted bioinformatics analyses to separate mtDNA reads from nuclear DNA reads for assembly and annotation of mitogenomes (see below).

After extraction, all DNA samples were treated with RNase A (Invitrogen) at 37 °C for 30 min and then 70 °C for 10 min. DNA samples were further purified using SPRselect Beads (Beckman Coulter). Quality and quantity of DNA were measured using a Nanodrop and a Qubit fluorometer (Invitrogen).

### Preparation, amplification and sequencing of Illumina libraries

A 150 nucleotide (nt) × 2 paired-end library for each sample was prepared (KAPA Hyper Prep Kit, KAPA Biosystems, www.kapabiosystems.com). Each DNA sample was barcoded by an adaptor. Sequencing the libraries was performed on the Illumina NextSeq500 platform at the UNM Biology Department’s Molecular Biology Facility (http://ceti.unm.edu/core-facilities/molecular-biology.html).

### Assembly and annotation of mitogenomes

Two methods were used to assemble mitochondrial genomes, the semi-reference based assembly using MITOBIM and de novo assembly using SPAdes^52^. MITOBIM is a tool developed to recursively find reads mapped to related reference mitogenomes and uses these reads to build the targeted mitogenome. The published mitogenome of M line *B. glabrata* was used as a reference^24^. In addition, de novo assembly using SPAdes was also conducted. The longest contig with BLASTN e-value < 10^−5^ against the reference mitogenome was selected to assemble the new mitogenome. Mitogenomes based on the two methods were aligned and manually checked for consistency. To check read support consistency, reads were mapped to final assembled mitogenomes and visualized using Integrated Genome Viewer^53^.

Mitogenome annotation was done using MITOS2 that includes the latest protein identification model^54,55^. We used two references in MITOS2, RefSeq63 Metazoa and RefSeq81 Metazoa, to annotate the mitogenomes for verification and confirmation, especially if one version did not predict all genes. For other criteria, default settings were applied (E-value exponent: 2; final maximum overlap: 50; fragment quality factor: 100). Moreover, we have re-checked mitogenome sequences manually using the ExPASY translation tool (http://web.expasy.org/translate/), which enabled us to see the correct reading frames of protein coding genes and to identify potential start and stop codons in the flanking regions of predicted genes. Based on annotation features, multiple circular mitogenome maps were drawn using the R package Circlize^56,57^.

### Assembly and annotation of nuclear rDNA

Two methods, de novo assembly and mapping reads to reference *B. glabrata* BBO2 rDNA, were used for assembly. Annotation of gene segment of nuclear rDNA that consists of 18S, ITS1, 5.8S, ITS2, and 28S was determined based on BBO2 rDNA sequence (http://biology.unm.edu/Biomphalaria-Genome/rDNABg.html).

### Genetic and phylogenetic analyses

Sequence alignments and percent identity of nucleotides (nt) and amino acids (aa) were determined using Clustal Omega^58^ (https://www.ebi.ac.uk/Tools/msa/clustalo/). Heat-maps of pair-wise sequence identities were generated using the R package ggplot2^59^. Evolutionary rate including synonymous, non-synonymous substitution rates and their ratio were calculated using KaKsCalculator^60^. The line plot chart for mitochondrial genes Ka/Ks comparisons among species was drawn using R package ggplot2^59^. All the intermediate data organization and filtering were done with in-house bash and Perl scripts and Microsoft Excel.

Phylogenetic analyses were performed using MEGA7^61^. Alignments were conducted using ClustalW integrated in MEGA7. Three methods were used for construction of trees, Maximum Likelihood (ML), Neighbor-Joining (NJ) and Minimum Evolution (ME). To evaluate the reliability of phylogenetic relationships, 1,000 bootstrap replications were applied in the three methods.

### Accession Codes

GenBank accession numbers of the mitogenomes for *B. pfeifferi*, *B. sudanica*, *B*. *choanomphala*, and G72M and G16BS90 lines of *B*. *glabrata*, are MG431962, MG431963, MG431964, MG431965, and MG431966, respectively. The Genbank accession numbers of rDNA for the five specimens (in the same order) are MG461588, MG461589, MG461590, MG461591, and MG461592, respectively.

## Electronic supplementary material

Supplementary dataset 1

Supplementary dataset 2

Supplementary information
