# Supplementary material for: Complete mitochondrial and rDNA complex sequences of important vector species of Biomphalaria, obligatory hosts of the human-infecting blood fluke, Schistosoma mansoni
Source: Sci Rep. 2018 May 9;8:7341. doi: 10.1038/s41598-018-25463-z (PMC5943310; doi:10.1038/s41598-018-25463-z)
Supplement: Supplementary file 3 — Supplementary information [file 41598_2018_25463_MOESM3_ESM.pdf]

Complete mitochondrial and rDNA complex sequences of important vector species of *Biomphalaria*, obligatory hosts of the human-infecting blood fluke, *Schistosoma mansoni*

Si-Ming Zhang, Lijing Bu, Martina R. Laidemitt, Lijun Lu, Martin W. Mutuku, Gerald M. Mkoji, & Eric S. Loker

Supplementary Figure 1. The positions related to each element of rDNA cassette are provided in **A**. The nt identity is presented in **B** (28S), **C** (ITS1 (red) and ITS2 (blue)), and **D** (two ITSs combined).

Supplementary Figure 2. The phylogenetic trees were built using the three methods (ML, NJ and ME) and three types of sequences (ITS1, ITS2 and ITS1 and 2 combined). For *B. tenagophila* (BT), ITS1 and 2 were from GenBank accession number (AY030388). For *Physella acuta*, ITS1 and 2 were from KF316327 and KF316328, respectively. Please note that ITS1 and 2 sequences were not available in *Radix*, so sequences of ITS1 and 2 from *Physella acuta* were used as outgroup.

Supplementary Figure 3. The NJ tree based on partial mtCoxI sequences currently available in GenBank shows the relationships of *B. sudanica* or *B. sudanica*-like snails in Africa. Since there are a large number of sequences from *B. sudanica*-like snails collected from Lake Victoria, only few sequences were randomly selected. All mtCoxI sequences from other localities were used if they could be aligned to the sequences of *B. sudancia* derived from Lake Victoria (Stanley et al., 2011, 2014). The GenBank accession numbers starting with AF, DQ, EU, and HM indicate that data derived from publications of Campbell et al.[37], Jørgensen et al.[21], Plam et al.[45], and Stanley et al. [10,11], respectively. The mtCoxI sequences of *B. glabrata* G72 M line (Bg), *B. sudanica* (BS) and *B. choanomphala* (BC) were from present study. Geographical locations are provided at the end of the accession numbers.

Suppl Fig.1

A

	BP	BS	BC	G72M	G16BS
18S	1-1829	1-1829	1-1829	1-1829	1-1829
ITS1	1830-2397	1830-2379	1830-2378	1830-2390	1830-2390
5.8S	2398-2553	2378-2535	2379-2534	2391-2546	2391-2546
ITS2	2554-2975	2536-2972	2535-2971	2547-2971	2547-2971
28S	2976-6755	2973-6749	2972-6748	2972-6765	2972-6765

B

	BP	BS	BC	G72M	G16BS
BP		99.79	99.79	99.76	99.76
BS			100	99.84	99.84
BC				99.84	99.84
G72M					100
G16BS					

C

	BP	BS	BC	G72M	G16BS
BP		86.11	86.26	86.64	87.00
BS	91.99		99.82	91.26	91.26
BC	91.92	99.77		91.06	91.06
G72M	90.74	92.91	92.91		99.11
G16BS	90.74	92.91	92.91	100	

D

	BP	BS	BC	G72M	G16BS
BP		90.81	90.90	87.89	87.89
BS			99.80	92.08	92.08
BC				91.97	91.97
G72M					99.49
G16BS					

Suppl Fig.2

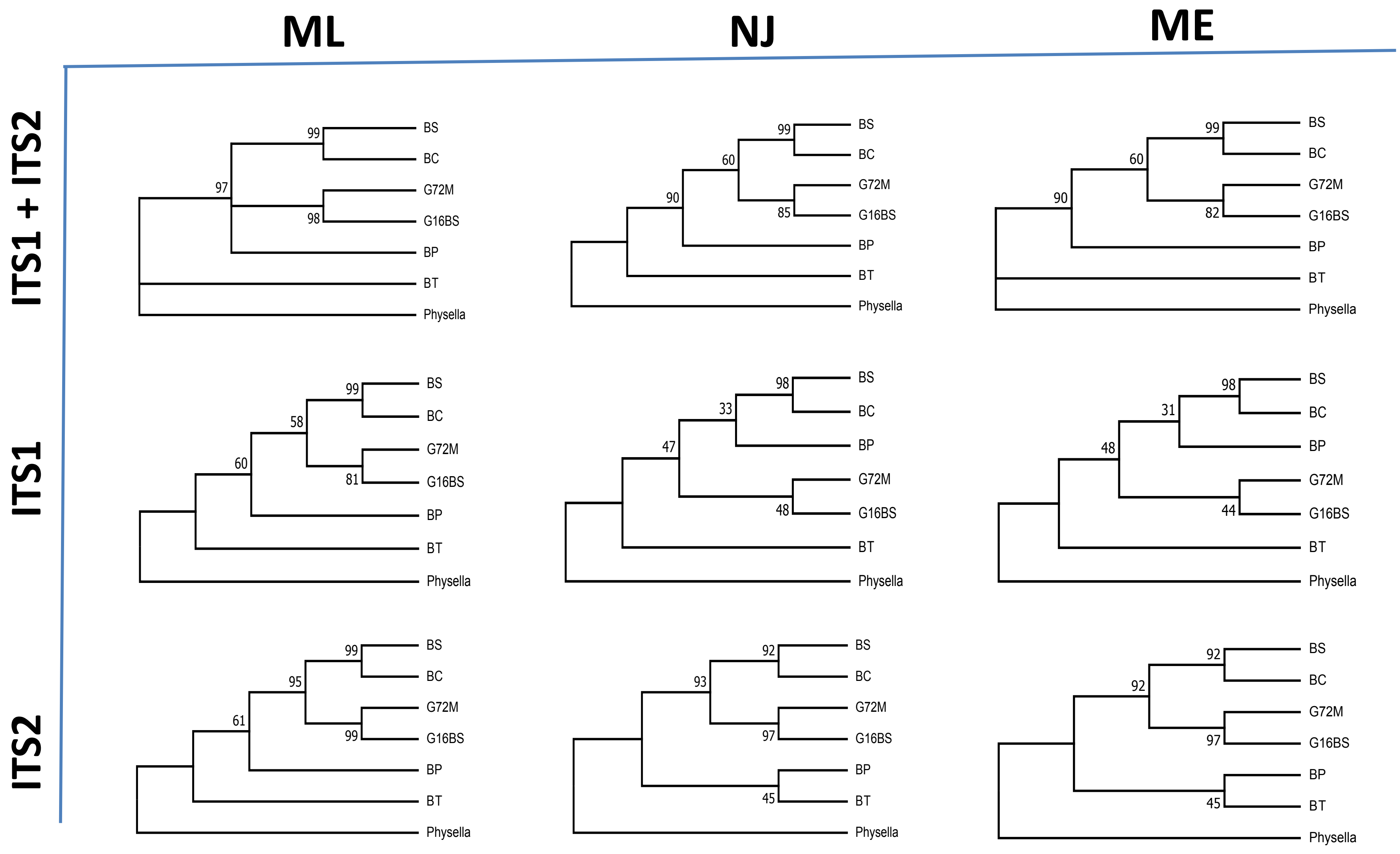
ML
NJ
ME
ITS1 + ITS2
ITS1
ITS2
BS
BC
G72M
G16BS
BP
BT
Physella
99
97
98
60
90
85
82
58
81
33
47
48
31
44
95
61
92
93
97
45

Suppl Fig.2

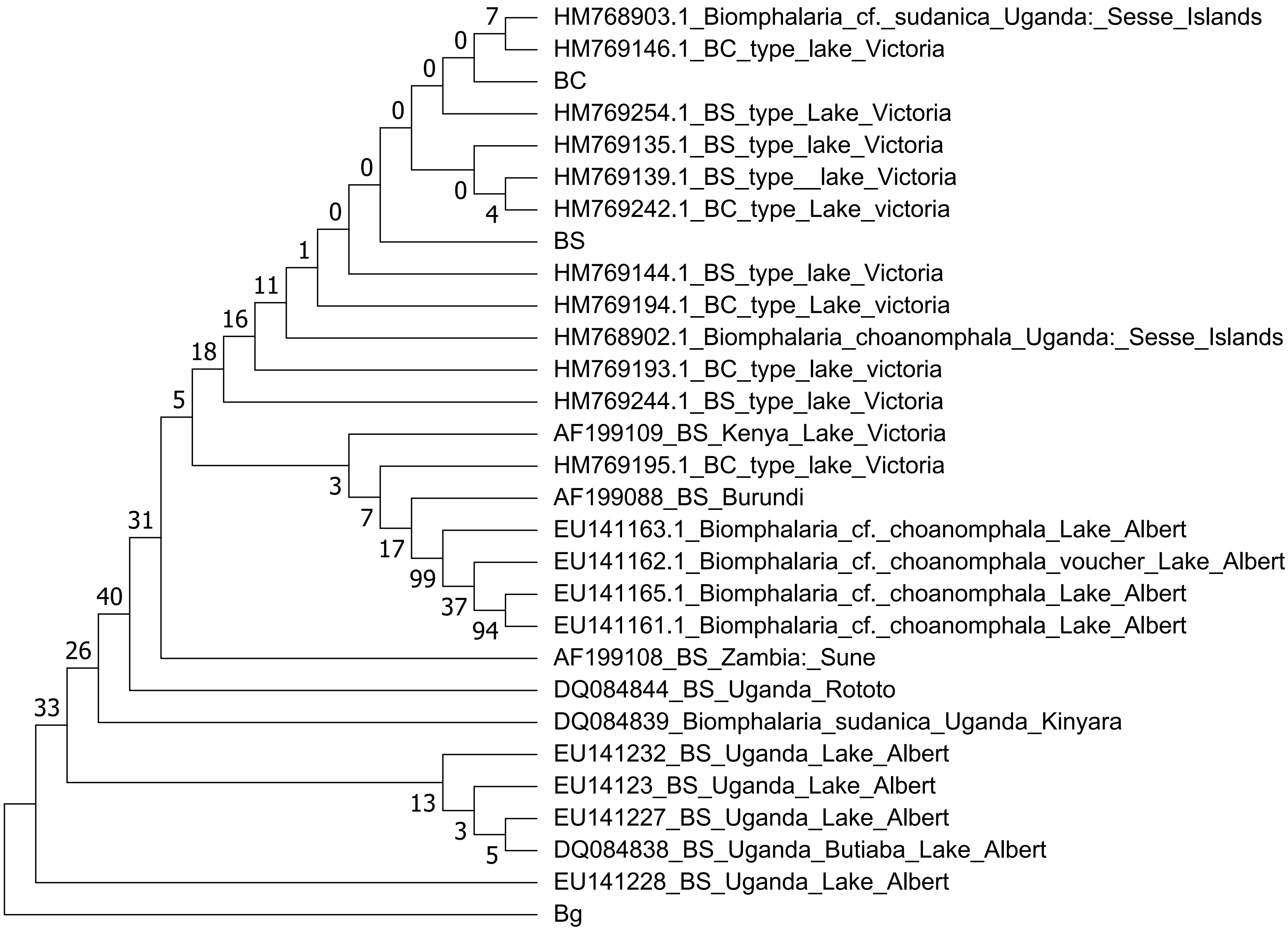
HM768903.1_Biomphalaria_cf._sudanica_Uganda:_Sesse_Islands
HM769146.1_BC_type_lake_Victoria
BC
HM769254.1_BS_type_Lake_Victoria
HM769135.1_BS_type_lake_Victoria
HM769139.1_BS_type__lake_Victoria
HM769242.1_BC_type_Lake_victoria
BS
HM769144.1_BS_type_lake_Victoria
HM769194.1_BC_type_Lake_victoria
HM768902.1_Biomphalaria_choanomphala_Uganda:_Sesse_Islands
HM769193.1_BC_type_lake_victoria
HM769244.1_BS_type_lake_Victoria
AF199109_BS_Kenya_Lake_Victoria
HM769195.1_BC_type_lake_Victoria
AF199088_BS_Burundi
EU141163.1_Biomphalaria_cf._choanomphala_Lake_Albert
EU141162.1_Biomphalaria_cf._choanomphala_voucher_Lake_Albert
EU141165.1_Biomphalaria_cf._choanomphala_Lake_Albert
EU141161.1_Biomphalaria_cf._choanomphala_Lake_Albert
AF199108_BS_Zambia:_Sune
DQ084844_BS_Uganda_Rototo
DQ084839_Biomphalaria_sudanica_Uganda_Kinyara
EU141232_BS_Uganda_Lake_Albert
EU14123_BS_Uganda_Lake_Albert
EU141227_BS_Uganda_Lake_Albert
DQ084838_BS_Uganda_Butiaba_Lake_Albert
EU141228_BS_Uganda_Lake_Albert
Bg
7
0
0
0
0
0
4
0
1
11
16
18
5
3
7
17
99
37
94
31
40
26
33
13
3
5
